# 341 Repeats Is Not Enough for Methylation in a New Fragile X Mouse Model

**DOI:** 10.1523/ENEURO.0142-22.2022

**Published:** 2022-09-06

**Authors:** Steven Colvin, Nick Lea, Qiangge Zhang, Martin Wienisch, Tobias Kaiser, Tomomi Aida, Guoping Feng

**Affiliations:** 1 Department of Biology, Massachusetts Institute of Technology, Cambridge, Massachusetts 02139; 2 Department of Brain and Cognitive Sciences, McGovern Institute for Brain Research, Massachusetts Institute of Technology, Cambridge, Massachusetts 02139; 3 Stanley Center for Psychiatric Research, Broad Institute of MIT and Harvard, Cambridge, Massachusetts 02142

## Abstract

Fragile X syndrome (FXS) is a leading monogenic cause of intellectual disability and autism spectrum disorders, spurring decades of intense research and a multitude of mouse models. So far, these models do not recapitulate the genetic underpinning of classical FXS—CGG repeat-induced methylation of the *Fmr1* locus—and their findings have failed to translate into the clinic. We sought to answer whether this disparity was because of low repeat length and generated a novel mouse line with 341 repeats, *Fmr1^hs341^*, which is the largest allele in mice reported to date. This repeat length is significantly longer than the 200 repeats generally required for methylation of the repeat tract and promoter region in FXS patients, which leads to silencing of the *FMR1* gene. Bisulfite sequencing fails to detect the robust methylation expected of FXS in *Fmr1^hs341^* mice. Quantitative real-time PCR and Western blotting results also do not resemble FXS and instead produce a biochemical profile consistent with the fragile X-associated premutation disorders. These findings suggest that repeat length is unlikely to be the core determinant preventing methylation in mice, and other organisms phylogenetically closer to humans may be required to effectively model FXS.

## Significance Statement

It is critical for the study of disease, and the translatability of findings into the clinic, that the model used exhibits close homology to the human condition. There remains uncertainty whether knock-in mouse models can replicate the core etiology of fragile X syndrome (FXS) and methylate the *Fmr1* gene. We therefore generated a new mouse line with a repeat size that far exceeds the established boundary for human methylation, and we report the continued absence of methylation. Our characterization of this line affirms that alternative models may be required for the comprehensive study of FXS, while these new mice may offer a valuable tool for the study of unmethylated fragile X-associated disorders.

## Introduction

Fragile X syndrome (FXS) is the most commonly inherited form of intellectual disability and leading monogenic cause of autism spectrum disorders (Hagerman et al., 2010; Lubs et al., 2012; Duy and Budimirovic, 2017), representing between 2–6% and 5–10% of all cases, respectively (Darnell and Klann, 2013). The affected gene, *FMR1*, has a CGG trinucleotide repeat in its 5′ untranslated region that is susceptible to expansions and contractions (Oberlé et al., 1991). While unaffected individuals typically harbor ∼30 repeats (De Rubeis et al., 2012), individuals with FXS are generally found to possess >200 repeats. At this size, the CpG-heavy sequence and its surrounding regions methylate (Maddalena et al., 2001), effectively silencing *FMR1* gene expression and eliminating the protein product FMRP (fragile X mental retardation protein; Oberlé et al., 1991). Curiously, a distinct clinical outcome emerges in the premutation range between 55 and 200 repeats: fragile X-related primary ovarian insufficiency (FXPOI) and/or the neurodegenerative fragile X-associated tremor/ataxia syndrome (FXTAS; Willemsen et al., 2011). Because of the presence of *FMR1* on the X-chromosome, FXS and FXTAS are disproportionately more prevalent in males (Crawford et al., 2001), with nearly all FXS males diagnosed with mild to severe intellectual disability (Hagerman et al., 2017).

Despite its disease burden, much remains unknown about *FMR1*. (1) It is still unclear precisely when or how the repeat tract expands, although it is established that the expansion occurs with the female oocyte (Martin and Bell, 1943; McMurray, 2010; Zhao and Usdin, 2018). (2) There is significant debate on the root cause behind the methylation of the locus and whether such a process can be reversed to restore cognitive function (Liu et al., 2018). (3) More challenging still, we do not yet have a complete picture of the targets and function of FMRP, though advances in identifying its signaling pathways and known interactions suggest that it plays diverse roles in RNA transport and translation (Bardoni and Mandel, 2002; Bassell and Warren, 2008; Bagni and Oostra, 2013; Darnell and Klann, 2013; Sethna et al., 2014; Richter and Zhao, 2021). And (4) the etiologies of FXPOI and FXTAS remain uncertain, though many independent lines of evidence suggest RNA toxicity is involved (Todd et al., 2013). With these and other lingering questions, FXS continues to be an area of intense research.

A number of attempts have been made to model FXS in mice. The earliest knock-out attempts yielded promising insights into the biology of FMRP (Dutch-Belgian Fragile X Consortium, 1994; Huber et al., 2002; De Rubeis et al., 2012; He and Portera-Cailliau, 2013; Jacquemont et al., 2014), yet the therapeutic predictions have so far largely failed to translate into the clinic (Scharf et al., 2015). Others have since sought to expand the mouse CGG repeat through transgenics or careful breeding (Bontekoe et al., 2001; Brouwer et al., 2007; Entezam et al., 2007; Berman et al., 2014), but no methylation has been observed even at 230 repeats. These latter studies also report a much lower intergenerational expansion/contraction rate than what is observed in humans. There is speculation that, perhaps in relation to this difference, the requisite length for methylation in mice might be even larger (Brouwer et al., 2007). Conversely, there is the possibility that these inconsistencies between human and mouse underlie fundamental differences in biological processes, and thus mice lack the construct validity to act as a model for FXS (Kaiser and Feng, 2015; Foote et al., 2016).

We sought to determine whether *Fmr1* methylation could be induced in mice through a massively expanded, patient-derived CGG repeat tract. To test this hypothesis, we generated a new transgenic mouse line possessing 341 repeats, *Fmr1^hs341^*, by replacing the native mouse sequence with a DNA fragment of expanded CGG repeats copied from a FXS patient-derived cell line. This CGG repeat size is larger than any fully characterized in the literature. We found that *Fmr1* failed to methylate in these animals, and instead discovered biochemical hallmarks of the premutation. Our results suggest that the molecular mechanisms and epigenetic factors regulating mouse *Fmr1* are distinct, and that thoroughly unraveling the causes and treatments for FXS may require modeling in more evolutionarily proximal species.

## Materials and Methods

All oligonucleotide sequences are presented in 5′ to 3′ orientation.

Individual animals are referenced as F<litter>.<pup>. For example, F33.3 and F33.4 refer to the third and fourth pups, respectively, of the 33rd litter of *Fmr1^hs341^*.

### Guide RNA design

**Figure 1.**
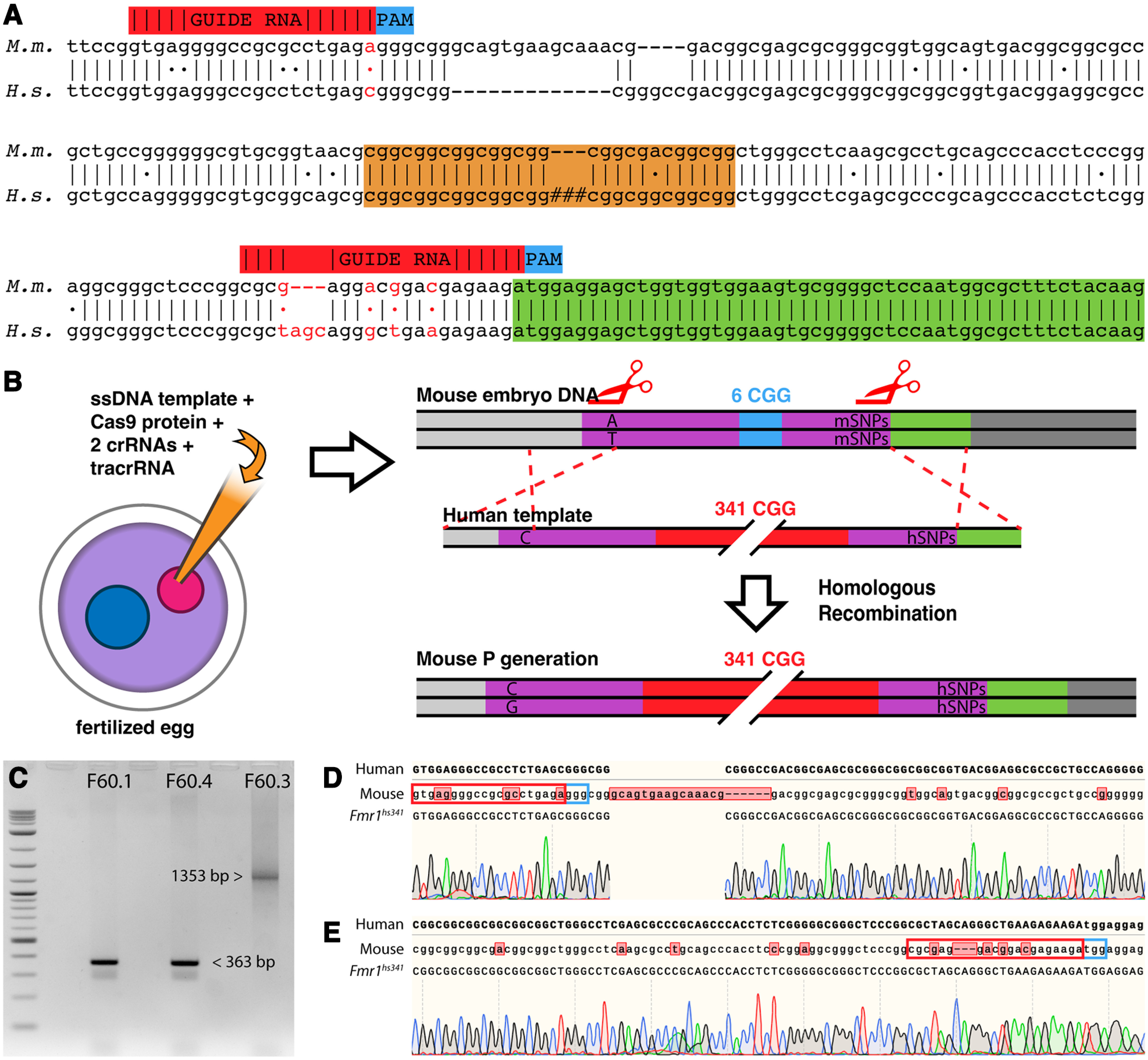
*FMR^hs341^* design and validation. ***A***, Sequence alignmnt. Annotated comparison between Mus musculus (M.m.) and Homo sapiens (H.s.) of the region surrounding the fragile X repeat tract. The repeat tract is highlighted in orange, with the # symbol representing variable CGG repeat length sizes in the human population. Guide RNA sequences and their associated PAM (Protospacer Adjacent Motif) sequence are indicated by their position above the M.m. sequence, with the SNPs incorporated by the template to prevent recutting shown in red (note: Cas9 cuts several nucleotides before the PAM sequence). The green box denotes the coding region of exon 1. Sequences are written 5′ to 3′. ***B***, Illustration of knock-in strategy. Embryos are injected with a mixture containing a single-stranded DNA template generated from human patient DNA, Cas9 protein, and two single-guide RNAs flanking the *FMR1* CGG repeat tract. The mouse embryo DNA is cut by Cas9 and repaired through homologous recombination with the patient mutation template. The exchange is irreversible because of SNPs in the corresponding guide sequence on the human allele. Purple, 5′ UTR; blue, CGG repeat tract of mouse; red, CGG repeat tract of human expansion; green, coding region of exon 1. ***C***, Confirmation of repeat size by gel electrophoresis. The *FMR1^hs341^* amplicon was predicted to be 1353 bp in length: 190 bp upstream, 1023 bp repeat tract (341 * 3), and 140 bp downstream. Lane 1 provides a DNA ladder (1 kb Plus DNA Ladder; catalog #N3200L, New England BioLabs), lanes 3 and 5 were identified as wild type, lane 7 was identified as *FMR1^hs341^*. ***D***, ***E***, Sanger sequencing of upstream and downstream regions, respectively. Red box denotes guide RNA recognition sequence; blue box denotes PAM sequence. The upstream region shows strong incorporation of the human template including several SNPs located 14 bp before the predicted cut site. Upstream sequencing penetrated up to 59 CGG repeats. The downstream region demonstrates precise integration of the human template, and sequencing penetrated up to 74 CGG repeats. Two nucleotides were manually annotated for the downstream sequence. Off-target traces can be found in Extended Data Figure 1-1.

Guides were designed using CRISPOR (Concordet and Haeussler, 2018). Guides were selected based on their proximity to the CGG repeats and whether the inserted human template sequence possessed one or more single-nucleotide polymorphisms (SNPs) from the mouse host genome, which protect the repaired knock-in allele from recognition by the guide RNA and subsequent recutting. The upstream guide was designed over an SNP at position 1, while the 3′ guide covered multiple SNPs between positions 8 and 18 (Fig. 1*A*; upstream guide: *GTGAGGGGCCGCGCCTGAGA*; downstream guide: *CGCGAGGACGGACGAGAAGA*).

10.1523/ENEURO.0142-22.2022.f1-1 Figure 1-1 **Off-target sequencing.** ***A–K***. Sequence traces of candidate off-target sites 1-12, respectively (note: off-target sites 11 and 12 are both found in ***K***). Matching off-target sequence is highlighted in blue. The *Fmr1^hs341^* F1 generation displays no sign of any off-target cutting. Download Figure 1-1, TIF file.

### CGG repeat template generation

Male patient DNA samples were purchased from the Coriell Institute (stock #LCL NA06852). The patient’s *FMR1* allele was previously characterized to have 341 repeats (Kwok et al., 2016). The allele was amplified with a reverse primer phosphorylated at its 5′ end to enable single-strand digestion and a forward primer protected by a phosphorothioated bond. These primers provided a homology arm length of 69 bp at the 5′ sequence and 56 bp at the 3′ sequence. DNA amplification was performed with a published PCR protocol (Hayward et al., 2016) and only slight modifications to the reagents: 50 mm Tris-HCl, pH 8.9, 22 mm (NH_4_)_2_SO_4_, 0.2% Triton X-100, 1.5 mm MgCl_2_, 0.8 μm forward primer, 0.8 μm reverse primer, 1.4 mm dGTP (deoxy-GTP), 1.4 mm dCTP (deoxycytidine triphosphate), 0.2 mm dATP (deoxy-ATP), 0.2 mm dTTP (deoxythymidine), 2% dimethylsulfoxide, 2.5 m betaine, ∼65 ng patient DNA, and 1 U Phusion DNA Polymerase (catalog #M0530L, New England BioLabs) in a 25 μl reaction volume (forward primer: G*CCCTTGGCCTCAGTCAGTCAGGCGCTGGGGAGCGTTTCGGTTTCACTTCCGGTGGAGG; reverse primer: p*TACCTTGTAGAAAGCGCCATTGGAGCCC* (where “*” is a phosphorothioate bond, and “p” is a phosphorylated primer).

The amplified DNA product was purified with the DNA Clean & Concentrator-5 Kit (ZYMO RESEARCH) and eluted with two applications of 12 μl of pure water warmed to 60°C. The purified product was subsequently digested to single strand with the Guide-it Long ssDNA Production System (Takara). Lastly, the product was purified with the NucleoSpin Gel and PCR Clean-Up Kit eluted with one application of 15 μl of 70°C water incubated for 5 min followed by a second application of 22.5 μl of 70°C water.

### Mouse embryo injection

The injection mixture was prepared with modifications on Aida et al. (2015). An initial volume of water was pipetted on ice to achieve a final volume of 30 μl: 0.625 μm 5′ guide, 0.625 μm 3′ guide, and 1.25 μm trans-activating CRISPR RNA (tracrRNA) were added to the water, incubated for 5 min at 5°C, and were left at room temperature for 10 min; 0.36 μl of Cas9-NLS (catalog #M0646M to Cas9-NLS, New England BioLabs) was added, and the mixture was heated to 37°C for 15 min. Lastly, 300 ng of the single-strand template product was added. The injection itself was performed by an on-site transgenic facility, as previously described (Wilde et al., 2021).

### Animal work

All animal procedures were performed in accordance with the Massachusetts Institute of Technology animal care committee regulations. Quantitative real-time PCR (qRT-PCR) was performed at postnatal day 21, while Western blotting and behavior experiments were performed at between 42 and 72 weeks of age. Sibling male mice were group housed and used as control and experimental groups.

### Genomic DNA preparation

Mouse genomic DNA was extracted from mouse tail tissue samples with the Machery–Nagel NucleoSpin Tissue Kit following the standard protocol eluting in 60 μl of 60°C warmed water.

### Genotyping

Genotyping was performed identical to the template generation above with distinct genotyping primers (forward primer: *CGGGTCACGTGACATCGTTTGACTGTTTACAGG*; reverse primer: *CCTGTCCGGTAGCCGGTTACCTTGTAGAAAGC*). The forward primer is outside the template sequence, but because of the complexity of amplifying this sequence the reverse primer partially overlaps the 3′ homology arm.

### On-target and off-target analysis

On-target and off-target editing were verified on F1 dames. The fragile X locus was amplified with the above genotyping protocol and sequenced by GENEWIZ for on-target confirmation. Candidate off-target sites were identified with the CRISPOR software (Concordet and Haeussler, 2018) under the criteria that the site had three or fewer mismatches from the guide target, or that the site had four mismatches from the guide target and was located within an exon. The search yielded 12 sites located on 11 unique loci. Each site was amplified with BioTaq (Meridian Bioscience) using the recommended protocol and sequenced by GENEWIZ (Table 1, primers). Trace files were aligned by SnapGene to *in silico* assemblies.

**Table 1:** Off-Target primers: primers for amplifying and sequencing candidate off-target sites

Off-target site	Guide	Chromosome	Gene	Mismatch	Forward primer	Reverse primer	Sequencing primer
1	Upstream	9	Trank1	3	AGGGCCCTTAGCATTTTTGC	AGCCAGCTGCAAGGAAACTC	GGATGTCTCCCTCTATGCAGTG
2	Upstream	7		3	GTACTGTCTTGGTGAGGGATTG	CCCAACAGAAGGCAGAGTAAG	CTCATACCATACCGTGCAG
3	Upstream	1		3	CAGAGGCAGGTGGATTTCTAAG	CGGTTCTAGGATTGCTGTTCTC	TTCTCCATCACCATGCTGTG
4	Upstream	18		3	AGTTGCATCTCACAGTTCCTATC	CTATGGGCCGGCTTCTATTATG	GTAAGCTGATCTCTCCGATC
5	Upstream	12		3	GAATACAGAGCTGAGGGAATGG	GGAGAACATGAGAGCTGGATATG	CAGTAGTAGAGCCATCCTTAC
6	Upstream	17		3	GATTATCAGCAGGGCTAGGATG	AGAGAGCATTGTGGGAATGAG	CCTTTAGCTCTGGGAGGTTTC
7	Upstream	5		3	GGTTCAGTTGCTTCCCAGTT	CCCGAAAGCTTGATCGAAGAG	CTGAGGATCTAGAAGCACTG
8	Upstream	7		3	TTCTGTACGGTTGGCTTCTTC	GGTGAGTCATCTCAGCAATCTC	ACTTCAGGAGTCTTCTCTC
9	Upstream	9	Mcam	3	TGAGGGTAAGGAGAGGGTAAG	GTACCATAGGACTTGAGGAATGG	GTCGCTTGCTCTTACACAG
10	Upstream	4	Ctnnbip1	4	TGCCTCAGCCGGAAATAAG	ACACAGACACACAGACACATAG	CTAGGGTCTCAAGCCTTC
11	Downstream	7		3	CCACCTTACACTAGCCATGAAC	CAAGGGCAGGATTGGAAGATAC	AGAGACACAGAGGGACAAG
12	Downstream	7		3	CCACCTTACACTAGCCATGAAC	CAAGGGCAGGATTGGAAGATAC	AGAGACACAGAGGGACAAG

### Bisulfite sequencing

Ten microliters of genomic DNA from male mice was treated with bisulfite to deaminate unmethylated cytosines to uracil with the EpiMark Bisulfite Conversion Kit (New England BioLabs) following standard protocol. Bisulfite-treated DNA was eluted with 40 μl of elution buffer and was either used immediately or aliquoted and stored at −20°C. Four microliters of bisulfite-converted DNA was used in 25 μl PCR reactions with following conditions: 1× EpiMark Reaction Buffer, 0.2 μm dNTPs (deoxynucleotide triphosphate), 0.2 μm each primer, and 0.625 U of EpiMark Polymerase. Reactions were mixed and immediately transferred to a preheated thermocycler with the following program: (1) 95°C for 30 s; (2) 95°C for 20 s; (3) 57°C for 30 s; (4) 68°C for 15 s; (5) repeat steps 2–4 35 times; (6) 68°C for 5 min; and (7) hold at 4°C. Four replicates were performed per sample, with 2 μl of each PCR product used in a second round of PCR. One reaction was run on a 1% agarose gel to verify correct amplification, while the remaining three reactions were pooled and column purified using the Machery-Nagel Clean and Concentrator-5 Kit following standard protocol and eluting in 5 μl of elution buffer. Two microliters of this purified PCR product was used as insert DNA in the TOPO TA Ligation Cloning Kit following the standard protocol. Plasmid DNA was miniprepped with ZR Plasmid Miniprep-Classic Kit (ZYMO RESEARCH) following the standard protocol. Insert sequences were sequenced by GENEWIZ with M13 (−20) forward and M13 reverse primers. Artificially methylated control samples were treated with M.SssI (catalog #M0226, New England BioLabs) according to manufacturer instructions and incubated for 1 or 2 h before bisulfite exposure (forward primer: *TTTTGATATTTTGAGGTAGGTATTT*; reverse primer: *CTAACTAACTAAAACCAAAAACTCC*).

### Dissection

Brain tissue of male mice was dissected and separated into corresponding brain regions before being snap frozen in liquid nitrogen and stored at −80°C.

### qRT-PCR

Brain tissue of male mice was processed with the Aurum Total RNA Mini Kit (BIO-RAD) aided by passing the sample through a 20 gauge needle after homogenization. The final elution volume was 60 μl. The resulting RNA was reverse transcribed with the iScript Advanced cDNA Synthesis Kit (BIO-RAD) with a 5 min 27°C step preceding the 46°C reverse transcription. The final cDNA product was analyzed using the SsoAdvanced Universal SYBR Green Supermix (BIO-RAD) following standard protocol with *Gapdh* and *β-Actin* serving as reference genes on a CFX96 Touch Real-Time PCR Detection System (BIO-RAD; *Fmr1* forward primer: *GCTGAAGATGTCATACAGGTTCCACG*; *Fmr1* reverse primer: *CATTTTCAGCCTCAATCCTCACCCTC*; *Gapdh* forward primer: *GCCTTCCGTGTTCCTACC*; *Gapdh* reverse primer: *CCTCAGTGTAGCCCAAGATG*; *β-Actin* forward primer: *CTAAGGCCAACCGTGAAAAG*; *β-Actin* reverse primer: *ACCAGAGGCATACAGGGACA*).

### Western blotting

Three hundred microliters of ice-cold RIPA buffer (150 mm sodium chloride, 1% v/v Nonidet P-40, 0.5% w/v sodium deoxycholate, 0.1% w/v SDS, and 50 mm Tris-HCl, pH 8.0) with protease inhibitors (Complete Protease Inhibitor Tablets; catalog #05056489001, Roche) was added to 20–50 mg of brain tissue from male mice. Tissue was homogenized with Potter-Elvehjem tissue grinders washed twice with 200 μl of ice-cold RIPA buffer. Samples were sonicated by Omni-Ruptor 250 Ultrasonic Homogenizer over an ice bath for 10 pulses at 10% power and 70% OFF. Samples were shaken on an orbital shaker at 4°C for 1 h, then centrifuged at 12,000 rpm at 4°C for 20 min. Supernatant was removed and protein concentration was determined using Pierce BCA Kit following the standard protocol. Samples were diluted to equivalent concentrations by adding ice-cold RIPA buffer. Laemmli sample buffer with 10% β-mercaptoethanol (BIO-RAD) was added to samples and boiled at 99°C for 5 min. Thirty micrograms of protein per well was loaded on 4–20% Mini Protean TGX precast gels and subjected to SDS-PAGE electrophoresis in Tris/glycine SDS buffer (25 mm Tris, 192 mm glycine, 0.1% SDS, pH 8.3) at 100 V for 90 min at 4°C. Proteins were transferred to nitrocellulose membranes at 4°C at 100 V for 60 min in Tris/glycine buffer with 20% methanol (25 mm Tris, 192 mm glycine, and 20% methanol v/v, pH 8.3). Membrane was air dried then washed in Tris-buffered saline (TBS; 50 mm Tris-HCl, 150 mm NaCl, pH 7.5) for 5 min with shaking on an orbital shaker. Membranes were blocked with 5% dry milk powder in TBS for 1 h at room temperature and washed three times for 5 min in TBS with 0.1% v/v Tween 20 (TBS-T 0.1%) while shaking. Membranes were incubated in a solution of 1:1000 rabbit anti-FMRP antibody (catalog #4317, Cell Signaling Technology) and 1:5000 mouse anti-GAPDH (6C5; catalog #SC-32233, Santa Cruz Biotechnology) in TBS-T 0.1% with shaking at 4°C overnight. Membranes were rewashed three more times before being incubated in a solution of 1:10,000 IRDye 800CW goat anti-rabbit IgG secondary antibody (LI-COR) and 1:10,000 IRDye 680RD goat anti-mouse IgG secondary antibody (LI-COR) in TBS-T 0.1% at room temperature for 1 h while shaking. Membranes were washed three more times, followed by three additional washes with TBS for 1 min. Membranes were then imaged on an Odyssey CLx (LI-COR). Intensities were confirmed to be in the linear range of detection compared with titration tests, and FMR1 signals were normalized to GAPDH loading controls.

### Behavior

#### Rotarod

Male mice were acclimated to handling for 1–2 min/d for at least 3 consecutive days before testing. Mice were transported to the testing room 30 min before testing. The rotarod apparatus (Med Associates) was set to 4 rpm, the mice were placed on the rods, and the rod was set to accelerate up to 40 rpm over 5 min. The time to fall was recorded. The mice were tested three times a day for 3 consecutive days, with at least 30 min between trials. The rotarod was cleaned with QUATRICIDE between runs. For each animal, rotarod testing was performed after all other behavioral tests.

#### Open field exploration

Male mice were acclimated to handling for 1–2 min/d for at least 3 consecutive days before testing. Mice were transported to the testing room 30 min before testing. Motor activity was measured in an open field arena (40 × 40 × 30 cm), which was indirectly illuminated at 60 lux, for 30 min. The apparatus was cleaned with QUATRICIDE before and between runs. Total distance traveled was quantified using an automated infrared detection system (Omnitech Digiscan, AccuScan Instruments). Raw data were extracted and analyzed using Microsoft Excel. For each animal, open field testing was conducted before all other behavioral tests.

#### Elevated zero maze

Male mice were acclimated to handling for 1–2 min/d for at least 3 consecutive days before testing. Mice were transported to the testing room 30 min before testing. The zero-maze apparatus was cleaned with QUATRICIDE before and after all tests. The elevated zero maze was indirectly illuminated at 100 lux in the open arms and 10–20 lux in the closed arms. The apparatus was video taped and tracked with Ethovision software (Noldus) for 10 min and scored using Observer software (Noldus). The test was initiated by placing the mouse in a closed area of the maze. The total distance traveled was measured for each animal. Data from animals that fell from the apparatus were omitted.

### Statistical methodology

Quantitative data from behavioral experiments were analyzed for estimation statistics. Normality could not be verified because of small sample sizes, so a median difference approach was used when comparing control and experimental datasets. The *p*-values were computed through a Kruskal–Wallis H test (Ho et al., 2019).

## Results

Earlier mouse models of FXS harboring CGG knockins fail to exhibit the classic molecular pattern of FXS: methylation of the *Fmr1* gene, transcriptional silencing, and absence of FMRP. To test whether larger repeat lengths may trigger these mechanisms, we generated a new FXS mouse line, *Fmr1^hs341^*. FXS patient DNA with 341 repeats was amplified using a modified PCR protocol (Hayward et al., 2016), followed by single-strand digestion, to produce a single-strand template comprising the patient repeat tract flanked by murine homology arms. We injected Cas9 protein, two CRISPR RNAs (crRNAs), tracrRNA, and the single-strand template into zygotes before pronuclei convergence, as illustrated in Figure 1*B*. These crRNA directed Cas9 to cut the endogenous mouse *Fmr1* on both sides of the CGG repeats, which the cell naturally repaired through homologous recombination using the patient-derived single-strand template (Mali et al., 2013).

Twenty-five percent of offspring (two of eight offspring) were heterozygous or mosaic female knockins. Transmission of the knock-in allele was verified in the F1 generation by both the length of the insertion and sequencing across the newly humanized region (Fig. 1*C–E*); these became the founders of the *Fmr1^hs341^* line. Candidate off-target sites were selected under the criteria that the site had three or fewer mismatches from the guide target, or that the site had four mismatches from the guide target and was located within an exon. All dames comprising the F1 generation were sequenced at each of these loci, and no off-target editing was detected (Extended Data Fig. 1-1). Although contractions were more common within the colony—8.1% of alleles with 341 repeats shrunk to <200 repeats within a single generation—*Fmr1^hs341^* expanded to >500 repeats within two generations. Nonetheless, compared with transmissions observed in humans (Reyniers et al., 1993; Nolin et al., 2003, 2015), the pattern and relative stability of intergenerational repeat length in *Fmr1^hs341^* and earlier mouse knock-in studies (Brouwer et al., 2007; Entezam et al., 2007; Zhao and Usdin, 2018) alludes to potential species differences in molecular genetics behavior at the fragile X locus.

**Figure 2.**
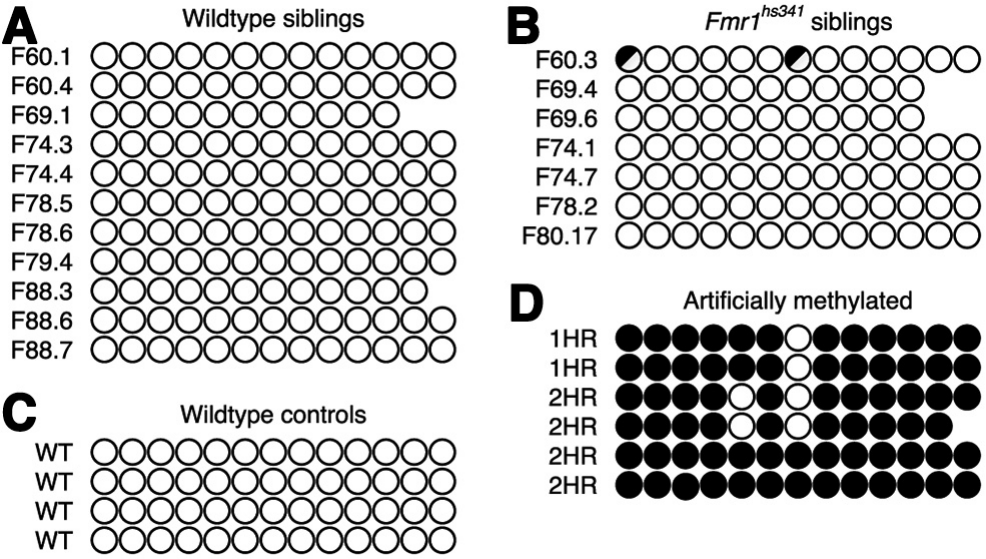
*FMR^hs341^* does not exhibit methylation. Bisulfite sequencing of male mouse DNA. Each row represents a single animal, and each circle represents one of the 13 CG cytosines within the sequencing region. Open circle, unmethylated; closed circle, methylated; split circle, partial methylation across multiple reads; no circle, missing in reads. All animals are referenced by F<litter>.<pup>. ***A***, Bisulfite sequencing of wild-type littermates. ***B***, Bisulfite sequencing of *Fmr1^hs341^* littermates. ***C***, Bisulfite sequencing of unrelated C57BL/6 mice. ***D***, Bisulfite sequencing of artificially methylated C57BL/6 mice, which served as a positive control that our methodology could detect methylated cytosines. Sequence traces can be found in Extended Data Figure 2-1.

**Figure 3.**
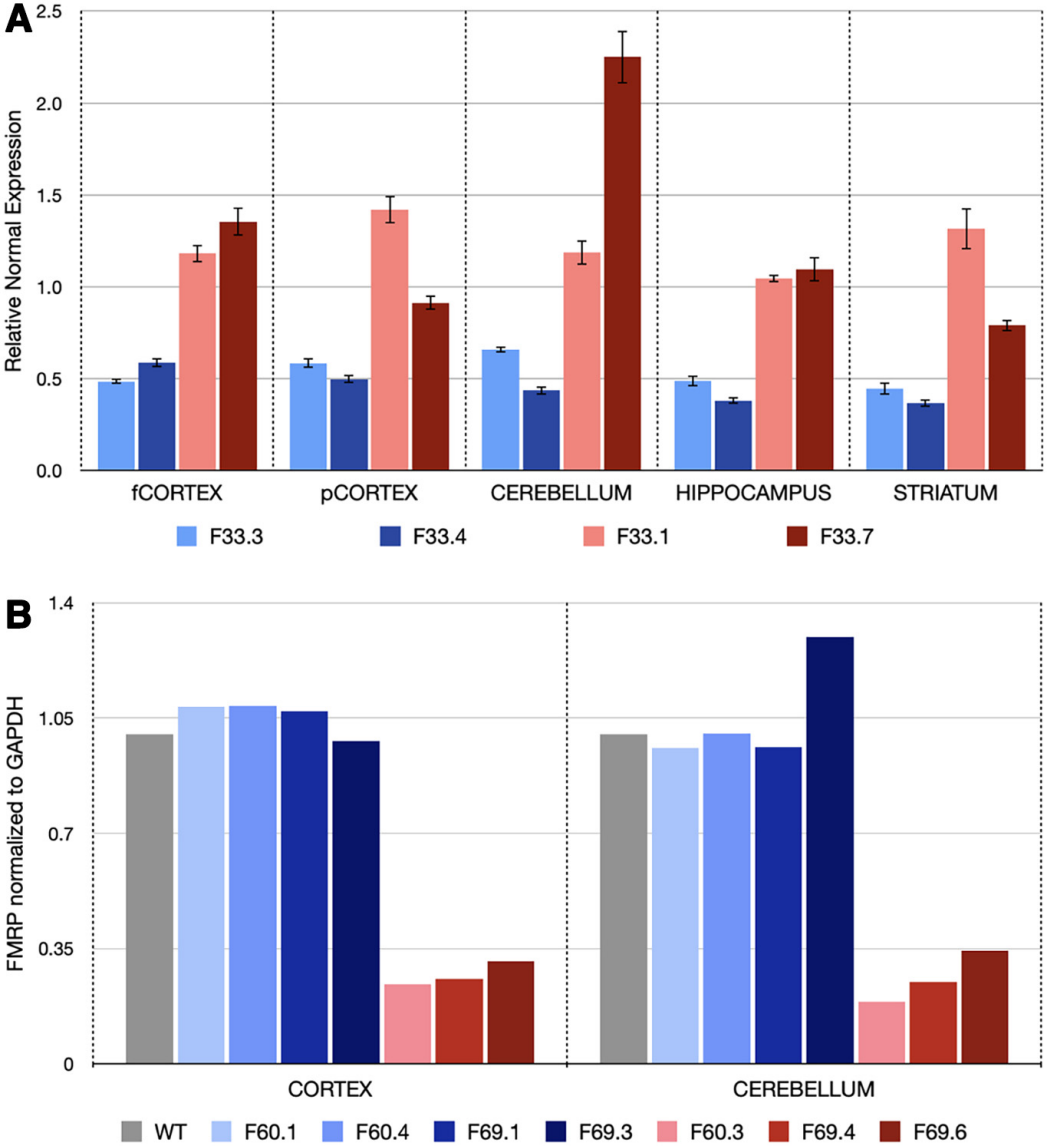
*FMR^hs341^* exhibits premutation molecular pathologies. All animals are referenced by F<litter>.<pup>. ***A***, qRT-PCR quantification. *Fmr1* mRNA is consistently higher in *Fmr1^hs341^* male mice (red) compared with their wild-type (blue) male sibling counterparts. Values are normalized to *Gapdh* and *Actin* expression across each brain region and presented as the mean ± SEM. fCORTEX, Frontal cortex; pCORTEX, posterior cortex. ***B***, Western blot quantification. FMRP is drastically reduced in *Fmr1^hs341^* (red) mice compared with wild-type (blue) siblings. Values are normalized to wild-type GAPDPH expression levels.

We wished to explore whether *Fmr1^hs341^* more closely resembled either diagnostic criteria for the full mutation (FXS) or the premutation (FXTAS/FXPOI). We first examined its methylation status, as *FMR1* is robustly methylated in FXS and unmethylated in both FXTAS/FXPOI and unaffected individuals (Naumann et al., 2009). Bisulfite sequencing of male *Fmr1^hs341^* mice in the region immediately upstream of the repeat tract (encompassing the putative transcriptional start site) showed no evidence of the extensive methylation found in FXS patients (Fig. 2). The absence of methylation in *Fmr1^hs341^* is consistent with human premutation and unaffected alleles, which suggests that *Fmr1* is still accessible for transcription in these mice. We therefore tested whether RNA levels are altered in *Fmr1^hs341^* mice through qRT-PCR. Our experiments found an average mRNA increase of 2.15-fold to 3.14-fold in *Fmr1^hs341^* male mice over wild-type (WT) male siblings in all brain regions tested (Fig. 3*A*). These results align with multiple studies in human premutation patients (Tassone et al., 2000a; Allen et al., 2004), unmethylated human full mutation patients (Tassone et al., 2000b), and mouse knock-in studies (Brouwer et al., 2007; Entezam et al., 2007), whereas methylated human patients with FXS produce little to no mRNA (Hagerman et al., 2017). We again conclude that *Fmr1^hs341^* fails to recreate a key feature of FXS and that the mouse *Fmr1* allele is unable to exhibit the same transcriptional dysregulation presented by FXS patients despite a similarly expanded repeat length. Although methylation underpins classical FXS and accounts for a majority of FXS cases, FXS is ultimately a result of the loss of function of FMRP, as demonstrated by patients diagnosed with FXS possessing a nonsense, missense, or frameshift mutation in *FMR1* yet normal CGG repeat length (Wells, 2009; Suhl and Warren, 2015; Tekendo-Ngongang et al., 2021). We therefore explored FMRP expression in *Fmr1^hs341^* and found our mutant male mice have appreciable, yet significantly depressed levels of FMRP (cortex, 25.6% of wild type; cerebellum, 24.7% of wild type; Fig. 3*B*). This incomplete reduction in FMRP is again consistent with the clinical appearance of the premutation and not the full mutation (Kenneson et al., 2001). The presence of *Fmr1* mRNA and FMRP verify that knockin of *Fmr1^hs341^* retains transcriptional and translational capabilities at the *Fmr1* locus. Yet overall, these findings extend those of other *Fmr1* repeat expansion mouse models and demonstrate that even when repeat length is expanded well into the full mutation range, the *Fmr1* locus fails to methylate or recapitulate other correlates of FXS.

10.1523/ENEURO.0142-22.2022.f2-1 Figure 2-1 Bisulfite sequencing traces. Representative sequence traces of bisulfite analysis. Sequences are aligned to the wild-type sequence where potentially protected cytosines are denoted with an uppercase C. ***A***, Wild-type sibling F88.6 shows no sign of methylation. ***B***, *Fmr1^hs341^* F80.17 shows no sign of methylation. ***C***, Wild-type mouse control shows no sign of methylation. ***D***, Artificially methylated mouse DNA (2 h incubation) demonstrates successful methylation. Download Figure 2-1, TIF file.

Despite lacking the molecular features of FXS, the *Fmr1^hs341^* line may still offer an important resource for FXTAS. Loss of motor skills is highly prevalent among individuals with FXTAS (Hall et al., 2014), so we therefore sought to provide a preliminary characterization for a small cohort of the *Fmr1^hs341^* line (Table 2, Extended Data Table 2-1). *Fmr1^hs341^* mice failed to exhibit any discernible difference in motor abilities and locomotion, performing equally well at the rotarod task as their wild-type counterparts (day 3: 141.5 ± 11.6 mean ± SEM, wild type: *n* = 11; 166.4 ± 17.0, *Fmr1^hs341^*: *n* = 9; *p* = 0.239*^c^*) and traveling similar distances in both the open field test (3161.2 ± 407.0 WT: *n* = 11, 3420.7 ± 289.1; *Fmr1^hs341^*: *n* = 9; *p* = 0.342*^d^*) and elevated zero maze (2346.0 ± 280.4 WT: *n* = 10, 2638.0 ± 342.2; *Fmr1^hs341^*: *n* = 5; *p* = 0.540*^e^*). Although further studies with larger sample sizes are required for a stronger consensus, our observations suggest that *Fmr1^hs341^* mice may also be an insufficient model for FXTAS despite a massively expanded CGG repeat length.

**Table 2:** Statistical table for behavioral experiments

Experiment	Data structure	Type of test	95% CI
*^a^* Rotarod day 1	Small *N* (nonparametric)	Kruskal–Wallis	−66.7, 34.0
*^b^* Rotarod day 2	Small *N* (nonparametric)	Kruskal–Wallis	−40.0, 75.7
*^c^* Rotarod day 3	Small *N* (nonparametric)	Kruskal–Wallis	−23.0, 90.0
*^d^* Open field distance	Small *N* (nonparametric)	Kruskal–Wallis	−1364.0, 962.0
*^e^* Zero plus distance	Small *N* (nonparametric)	Kruskal–Wallis	−1010.2, 1600.7

Graphical representations of results can be found in Extended Data Table 2-1.

10.1523/ENEURO.0142-22.2022.tab2-1 Table 2-1 ***FMR^hs341^* does not appear to cause motor deficits.** Estimation statistics for behavioral data. All mice were males between 12 and 15 months of age, and age-matched controls were used whenever wildtype siblings were not available. Rightmost data point (FM, full mutation minus NM, no mutation or ∆) represents difference in the medians for effect size. Weighted vertical line indicates 95% confidence interval. Filled curve reflects sampling-error distribution. Generated through Ho et al., 2019 (available at https://www.estimationstats.com/ at time of publication). NM (blue): wildtype; FM (orange): *Fmr1^hs341^*. ***A–C***. Time to first fall on the rotarod for mice across three consecutive days compared to wildtype littermates (*p*-values for: day 1 = 0.252; day 2 = 0.594; day 3 = 0.239) [NM: *N* = 11; FM: *N* = 9]. ***D***. Total distance traveled in the open field arena compared to wildtype littermates (*p*-value = 0.342) [NM: *N* = 11; FM: *N* = 9]. ***E***. Total distance traveled in the elevated zero maze apparatus compared to wildtype littermates (*p*-value = 0.540) [NM: *N* = 10; FM: *N* = 5]. Download Table 2-1, TIF file.

## Discussion

We have created a new mouse model of FXS possessing 341 CGG trinucleotide repeats, *Fmr1^hs341^*, far exceeding the ∼200 repeats often cited as the benchmark for FXS in human patients. This is the first such model to be derived through a nonvector approach by using PCR-based template generation, and the first to attain such a high repeat number in the first generation rather than through selective breeding.

Despite the outsized CGG repeat length, the molecular signature of *Fmr1^hs341^* shares little with the FXS full mutation. We failed to observe methylation of the *Fmr1* locus or complete loss of *Fmr1* RNA and FMRP, as expected of the full mutation. These findings reinforce a requirement for methylation to accurately model the phenotypes expected of FXS.

Several studies of knock-in mouse models for FXTAS have reported a correlation between repeat length and severity of phenotype (Brouwer et al., 2008; Diep et al., 2012; Ludwig et al., 2014). The large repeat length of *Fmr1^hs341^* may therefore expose clearer FXTAS phenotypes than previous models and enable more robust measurement of therapeutic interventions, though further validation is required. A strong indicator of construct validity would be the presence of intranuclear inclusions—a hallmark of FXTAS (Greco et al., 2002, 2006; Wenzel et al., 2010). Yet although these unmethylated *Fmr1^hs341^* mice experience molecular perturbations that closely resemble the FXTAS/FXPOI premutation (elevated *Fmr1* transcript levels and reduced FMRP protein), they do not appear to exhibit any clear motor defects expected of FXTAS. A larger behavioral cohort may better determine whether *Fmr1^hs341^* displays any neurophysiological symptoms of FXTAS; however, existing data on motor deficits in knock-in mouse models of FXTAS are limited and occasionally conflicting (Van Dam et al., 2005; Qin et al., 2011; Hunsaker, 2013; Foote et al., 2016; Haify et al., 2020). It is worth noting that FXTAS typically emerges after 50 years of age, so it is possible that these neurodegenerative symptoms will appear in *Fmr1^hs341^* mice of a more advanced age than used in this study (see “When are mice considered old?” from The Jackson Laboratory; https://www.jax.org/news-and-insights/jax-blog/2017/november/when-are-mice-considered-old).

The immutable epigenetic profile of the mouse *Fmr1* begs the question whether there may be distinct molecular factors at play in humans that are not present in mice. A truer question may be: where is the boundary of homology for these two species when studying FXS? It is important to clarify that these results do not suggest any opinion on whether the roles of FMRP itself are unique in the mouse (Denman and Sung, 2002), only that caution is advised when comparing these systems. These species differences may preclude development of translatable therapeutics in mice (Evans-Galea et al., 2013); therefore, there is greater need for rigorous validity testing and the production of models that are capable of accurately recapitulating the molecular, phenotypic, and behavioral symptoms of FXS. Nonhuman primates may be the next candidate because of their evolutionary closeness.

## Synthesis

Reviewing Editor: Deanna Smith, University of South Carolina Decisions are customarily a result of the Reviewing Editor and the peer reviewers coming together and discussing their recommendations until a consensus is reached. When revisions are invited, a fact-based synthesis statement explaining their decision and outlining what is needed to prepare a revision will be listed below. The following reviewer(s) agreed to reveal their identity: Laurens Bosman, Jeanne Loring.

Synthesis

Both reviewers and I feel the findings of this study are novel, suggesting fundamental differences in how mouse and human cells handle CGG repeats, and are thus of relevance to a large number of readers. The main findings of this manuscript are at the molecular level, and this is important new information. However, the behavioral experiments do not probe any specific feature of FXS. Pleas focus on the molecular findings and submit the behavioral data added as supplementary findings to show that this new mouse line has no gross motor phenotype. The authors hypothesize that their mice might resemble FXTAS more than FXS, and FXTAS also prominently includes motor symptoms. Examination of nuclear inclusions could further substantiate the comparison with FXTAS. Ultimately it will be important to use behavioral tests that are more relevant for the cognitive and social impairments observed in FXS (water maze, three chamber test, Y-maze etc) performed with a sufficiently large group size. It is also recommended that the authors include the following in supplemental data:

1. How is the genome of the transgenic line analyzed for correct targeting and lack of off-target changes? If sequencing was performed, then sequencing traces should be shown.

2. The method for determining the CGG repeat length is missing. Is it by capillary electrophoresis or genome mapping? Data on the CGG repeat length should also be shown, including different editing outcomes, such as the mice that had over 500 repeats within two generations.

3. It would be important to know the methylation status and transcription status of the mice with over 500 repeats.

4. It appears that the bisulfite sequencing was performed with Sanger sequencing. However, no traces are shown. The Sanger sequencing data should be included as supplementary data.

Below are all comments from each reviewer - please address:

Reviewer 1:

In general, I think this is an interesting study, and I appreciate the coupling between (epi)genetics and behavioral studies. However, I feel some parts could be improved. I have two general remarks and some more specific remarks.

1. Could the authors motivate the choice for these particular behavioral tests? As emphasized in their Introduction, FXS is characterized by autistic behavior and intellectual disabilities. Why were no tests for social behavior and/or cognitive abilities included in the battery of tests?

2. It is an interesting hypothesis that mice with an extreme repeat length, like used in this study, are (epi)genetically more related to FXTAS than to FXS. I would indeed expect that a severe impairment in motor function could be revealed by the behavioral tests employed, but all tests used are rather aspecific and I would not expect them to be able to pick up small differences, especially not with the small group sizes used. I can accept the conclusion that no major motor phenomenon is obvious in these mice, though. I was wondering, however, if the authors suspect the presence of FXTAS why they did not study the presence of nuclear inclusion, considered a hallmark for FXTAS.

Specific comments:

3. Methods (Open field testing):

Please indicate the illumination conditions during the open field testing as these can have an impact on anxiety.

4. Genomic stability:

At the bottom of page 15, the authors describe the relative stability of the repeat expansion in mice. This section, however, is not very clear to me. It is stated that contractions are “more common", with in 8.1% (of all mice or of the cases with contraction?) leading to repeat lengths <200. Does this contraction to <200 happen in one generation or in more? Sometimes (how often?) expansions to >500 occur in 2 generations. And afterwards? Do the expansions continue to grow, or shrink again, or stay stable? As this is, at least in my opinion, an important point, a figure (panel) devoted to this could be insightful.

Please indicate throughout the manuscript whether repeat length could affect the results or their interpretation.

5. Sex:

Please indicate at all relevant places, but at least in the Methods and the Figure legends, whether male or female mice were used.

6. Figure 3:

I assume the different codes refer to individual animals, but this is not explained in the Figure legend. Please consider to replace the bar graphs and SEM with another format, e.g. a dot plot, that gives a bitter insight into variations.

7. Figure 4:

n = 5 mice for a behavioral test is a very small number. No clear conclusions can be drawn.

8. Figure 4 (2):

I am afraid that the layout of Figure 4 is really confusing. Maybe using some symbols for the different tests could help, as well as grouping plots on the same behavioral test together (now: A = rotarod; B and D = open field; C and E = elevated plus maze).

9. Figure 4 (3):

What is the added value of the “sampling-error distribution”? I find this rather confusing; given the low number of replicates, I find this more distracting and confusing than elucidating.

Reviewer 2:

The mouse model that has been typically used to study Fragile X syndrome is a knockout of the Fmr1 gene. But the human disease is caused by silencing of FMR1 by methylation of the 5’ UTR caused by expansion of a CGG repeat sequence. In humans, more than 200 repeats generally causes methylation and silencing. Since mouse models are often poor mimics of a human disease, it is useful to determine whether modifying the mouse Fmr1 gene by insertion of a string of CGG repeats would create a more faithful model. This study used CRISPR-Cas9 technology to generate a transgenic line that carries 341 CGG repeats at the Fmr1 locus. This would be a sufficient number of repeats to induce methylation and silencing in the human. However, the expanded repeats did not cause methylation on the Fmr1 locus nor silence its transcription. The transgenic mice lack characteristics of FXS that had been reported in the Fmr1 knockout animals, but did show some similarities to the Fragile X-associated premutation disorders This is an important study for two reasons. First, it is a caution about putting too much faith in animal models as surrogates for human disease. Second, it highlights the species difference in DNA methylation, and raises questions about the differences in underlying mechanisms and regulation of DNA methylation in human and rodent, A shortcoming is the lack of certain key pieces of information about the methods and results. No supplementary materials were provided for this review, and should be added to include the following:

1. How is the genome of the transgenic line analyzed for correct targeting and lack of off-target changes? If sequencing was performed, then sequencing traces should be shown.

2. The method for determining the CGG repeat length is missing. Is it by capillary electrophoresis or genome mapping? Data on the CGG repeat length should also be shown, including different editing outcomes, such as the mice that had over 500 repeats within two generations.

3. It would be important to know the methylation status and transcription status of the mice with over 500 repeats.

4. It appears that the bisulfite sequencing was performed with Sanger sequencing. However, no traces are shown. The Sanger sequencing data should be included as supplementary data.

## Author Response

Dear Dr. Smith,

We would like to submit our revised manuscript, “341 Repeats is Not Enough for Methylation in a New Fragile X Mouse Model", for publication in eNeuro upon re-review.

We thank both yourself and the reviewers for your helpful comments and support of this work. We appreciate both the time and effort dedicated to the response, and have therefore strived to address each comment in turn. Significantly, we have added and emphasized data regarding on-target and off-target editing. We have also sought to eliminate confusion by focusing on the molecular findings and removing a majority of the behavior content. Your feedback has emphasized the critical points underlying the hypothesis, and we believe this new presentation of data will help draw attention to distinctions between animal models and humans, particularly with regard to Fragile X Syndrome.

Thank you for your consideration of our manuscript for publication in eNeuro.

Guoping Feng, PhD

Poitras Professor of Neuroscience

Massachusetts Institute of Technology

-

SUMMARIZED COMMENTS

1. Please focus on the molecular findings and submit the behavioral data added as supplementary findings to show that this new mouse line has no gross motor phenotype.

We greatly appreciate the suggestion to submit the behavioral data as supplementary findings. We were somewhat challenged with how to do so, as our interpretation of eNeuro publishing guidelines suggests Extended Data figures must support a main table or figure, which in turn must be referenced in the main text. We therefore reformatted the document to trim a majority of the behavioral text - eliminating anxiety measures. We further limited data presentation to the statistical table, and relocated the behavior graphs to Extended Data.

2. Examination of nuclear inclusions could further substantiate the comparison with FXTAS.

We thank the reviewers for their suggestion to explore nuclear inclusions, and agree that examination of inclusions would assist in validating the Fmr1hs341 line as a model for FXTAS. We hope to address this in a future study more specifically exploring and addressing features of FXTAS and FXPOI.

3. Ultimately it will be important to use behavioral tests that are more relevant for the cognitive and social impairments observed in FXS (water maze, three chamber test, Y-maze etc) performed with a sufficiently large group size.

We appreciate that our behavioral test size was of small n, and thank the reviewer for their comment. We have both relegated this data to Extended Data and highlighted the sample size for better transparency. We deliberated extensively on the selection of behavioral experiments and the intent of this article. Ultimately, we felt that our molecular data firmly established that the model was not FXS, and therefore felt it would be incorrect to apply behavioral assays for FXS to what is genetically a distinct disorder. We instead sought to focus on FXTAS-specific features . In retrospect, we recognize the impact of this decision on the report’s structure, and agree with the suggested placement of these experiments in Extended Data.

4. How is the genome of the transgenic line analyzed for correct targeting and lack of off-target changes? If sequencing was performed, then sequencing traces should be shown.

Thank you for noting these common concerns for gene editing, as they would certainly be asked by readers as well. On-targeting was confirmed by PCR amplification and sequencing of sites flanking the repeat tract, which verified insertion of the humanized sequence through SNPs introduced by our CRISPR strategy and repeat tract size. We have included these traces in Extended Data. We have also included sequencing results of candidate off-target sites and detailed the selection process for these sites within the manuscript.

5. The method for determining the CGG repeat length is missing. Is it by capillary electrophoresis or genome mapping? Data on the CGG repeat length should also be shown, including different editing outcomes, such as the mice that had over 500 repeats within two generations.

We apologize that the method for determining the CGG repeat length was not made clearer. We employed gel electrophoresis as the method for determining repeat length, a technique recommended by the Usdin laboratory in “A Set of Assays for the Comprehensive Analysis of FMR1 Alleles in the Fragile X-Related Disorders” (Hayward et al., 2016). We have included a gel in Figure 1 as well to this effect, which combined with the on-target analysis gives a more complete picture on the repeat length and editing outcome.

6. It would be important to know the methylation status and transcription status of the mice with over 500 repeats.

We thank the reviewer for raising this question, as we agree they would accentuate the point of our paper. Unfortunately, the two mice over 500 repeats were both female, and therefore were not valid for the experiments performed. We would be happy to add text informing readers of this information if requested.

7. It appears that the bisulfite sequencing was performed with Sanger sequencing. However, no traces are shown. The Sanger sequencing data should be included as supplementary data.

We thank the reviewer for the observation, and have added representative Sanger sequencing traces to the Extended Data.

-

REVIEWER 1

1. Could the authors motivate the choice for these particular behavioral tests? As emphasized in their Introduction, FXS is characterized by autistic behavior and intellectual disabilities. Why were no tests for social behavior and/or cognitive abilities included in the battery of tests?

We thank the reviewer for raising this concern. We deliberated extensively on the selection of behavioral experiments and the intent of this article. Ultimately, we felt that our molecular data firmly established that the model was not FXS, and therefore felt it would be incorrect to apply behavioral assays for FXS to what is genetically a distinct disorder. We instead sought to focus on FXTAS-specific features . In retrospect, we recognize the impact of this decision on the report’s structure, and agree with the suggested placement of these experiments in Extended Data.

2. It is an interesting hypothesis that mice with an extreme repeat length, like used in this study, are (epi)genetically more related to FXTAS than to FXS. I would indeed expect that a severe impairment in motor function could be revealed by the behavioral tests employed, but all tests used are rather aspecific and I would not expect them to be able to pick up small differences, especially not with the small group sizes used. I can accept the conclusion that no major motor phenomenon is obvious in these mice, though. I was wondering, however, if the authors suspect the presence of FXTAS why they did not study the presence of nuclear inclusion, considered a hallmark for FXTAS.

We thank the reviewers for their suggestion to explore nuclear inclusions, and agree that examination of inclusions would assist in validating the Fmr1hs341 line as a model for FXTAS. We hope to address this in a future study more specifically exploring and addressing features of FXTAS and FXPOI.

3. Please indicate the illumination conditions during the open field testing as these can have an impact on anxiety.

We thank the reviewer for this observation, and have added the information to our methodology.

4. At the bottom of page 15, the authors describe the relative stability of the repeat expansion in mice. This section, however, is not very clear to me. It is stated that contractions are “more common", with in 8.1% (of all mice or of the cases with contraction?) leading to repeat lengths <200. Does this contraction to <200 happen in one generation or in more? Sometimes (how often?) expansions to >500 occur in 2 generations. And afterwards? Do the expansions continue to grow, or shrink again, or stay stable? As this is, at least in my opinion, an important point, a figure (panel) devoted to this could be insightful.

Please indicate throughout the manuscript whether repeat length could affect the results or their interpretation. |

This is a critical variable studied by a number of fascinating articles, and we thank the reviewer for noting its absence here. We have added language on the correlation between repeat length and phenotype to the discussion, and additionally referenced several articles for readers to explore the topic further.

Unfortunately, our females were often group-housed due to space constraints and therefore individual lineages could not always be determined. We were careful to only report statistics that could be analyzed across the entire cohort, and therefore cannot answer the additional questions posed. We agree this would be an interesting follow up, as there are groups that seek to study the mechanisms behind this very question.

5. Please indicate at all relevant places, but at least in the Methods and the Figure legends, whether male or female mice were used.

Thank you for identifying this missing critical information. We have noted the sex for all experiments and repeated this information across sections for clarity.

6. I assume the different codes refer to individual animals, but this is not explained in the Figure legend. Please consider to replace the bar graphs and SEM with another format, e.g. a dot plot, that gives a bitter insight into variations.

We thank the reader for their comments. It was an oversight not to detail this nomenclature in the text, and we have sought to improve understanding by including this information throughout the manuscript. We hoped that these codes would allow readers to examine the relationship between animals (e.g. siblings) as well as between experiments (bisulfite and expression) for a more thorough understanding of the data. For this reason, we greatly wish to maintain the current format of this figure if possible.

7. n = 5 mice for a behavioral test is a very small number. No clear conclusions can be drawn.

We appreciate that our behavioral test size was of small n, and thank the reviewer for their comment. We have both relegated this data to Extended Data and highlighted the sample size for better transparency.

8. I am afraid that the layout of Figure 4 is really confusing. Maybe using some symbols for the different tests could help, as well as grouping plots on the same behavioral test together (now: A = rotarod; B and D = open field; C and E = elevated plus maze).

We apologize to the reviewer that Figure 4 failed to provide clear information, and appreciate their bringing this to our attention. In light of other comments, we hope the simplifications made to this figure in the revision have improved its readability, and we welcome any further comments or suggestions for improvement.

9. What is the added value of the “sampling-error distribution”? I find this rather confusing; given the low number of replicates, I find this more distracting and confusing than elucidating.

We must again apologize for the formatting. The statistical methodology was performed to align with eNeuro’s move towards estimation statistics, and the figures were generated via a tool recommended by eNeuro for this purpose. We ideally would like to conform to those guidelines - provided the information is still legible. We welcome any further comments if the reformatted figure fails to bring clarify to the data.

-

REVIEWER 2

1. How is the genome of the transgenic line analyzed for correct targeting and lack of off-target changes? If sequencing was performed, then sequencing traces should be shown.

Thank you for noting these common concerns for gene editing, as they would certainly be asked by readers as well. On-targeting was confirmed by PCR amplification and sequencing of sites flanking the repeat tract, which verified insertion of the humanized sequence through SNPs introduced by our CRISPR strategy and repeat tract size. We have included these traces in Extended Data. We have also included sequencing results of candidate off-target sites and detailed the selection process for these sites within the manuscript.

2. The method for determining the CGG repeat length is missing. Is it by capillary electrophoresis or genome mapping? Data on the CGG repeat length should also be shown, including different editing outcomes, such as the mice that had over 500 repeats within two generations.

We apologize that the method for determining the CGG repeat length was not made clearer. We employed gel electrophoresis as the method for determining repeat length, a technique recommended by the Usdin laboratory in “A Set of Assays for the Comprehensive Analysis of FMR1 Alleles in the Fragile X-Related Disorders” (Hayward et al., 2016). We have included a gel in Figure 1 as well to this effect, which combined with the on-target analysis gives a more complete picture on the repeat length and editing outcome.

3. It would be important to know the methylation status and transcription status of the mice with over 500 repeats.

We thank the reviewer for raising this question, as we agree they would accentuate the point of our paper. Unfortunately, the two mice over 500 repeats were both female, and therefore were not valid for the experiments performed. We would be happy to add text informing readers of this information if requested.

4. It appears that the bisulfite sequencing was performed with Sanger sequencing. However, no traces are shown. The Sanger sequencing data should be included as supplementary data.

We thank the reviewer for the observation, and have added representative Sanger sequencing traces to the Extended Data.

## References

[B1] Aida T, Chiyo K, Usami T, Ishikubo H, Imahashi R, Wada Y, Tanaka KF, Sakuma T, Yamamoto T, Tanaka K (2015) Cloning-free CRISPR/Cas system facilitates functional cassette knock-in in mice. Genome Biol 16:87. 10.1186/s13059-015-0653-x 25924609 PMC4414275

[B2] Allen EG, He W, Yadav-Shah M, Sherman SL (2004) A study of the distributional characteristics of FMR1 transcript levels in 238 individuals. Hum Genet 114:439–447. 10.1007/s00439-004-1086-x 14758538

[B3] Bagni C, Oostra BA (2013) Fragile X syndrome: from protein function to therapy. Am J Med Genet A 161A:2809–2821. 10.1002/ajmg.a.36241 24115651

[B4] Bardoni B, Mandel J-L (2002) Advances in understanding of fragile X pathogenesis and FMRP function, and in identification of X linked mental retardation genes. Curr Opin Genet Dev 12:284–293. 10.1016/s0959-437x(02)00300-3 12076671

[B5] Bassell GJ, Warren ST (2008) Fragile X syndrome: loss of local mRNA regulation alters synaptic development and function. Neuron 60:201–214. 10.1016/j.neuron.2008.10.004 18957214 PMC3691995

[B6] Berman RF, Buijsen RA, Usdin K, Pintado E, Kooy F, Pretto D, Pessah IN, Nelson DL, Zalewski Z, Charlet-Bergeurand N, Willemsen R, Hukema RK (2014) Mouse models of the fragile X premutation and fragile X-associated tremor/ataxia syndrome. J Neurodev Disord 6:25. 10.1186/1866-1955-6-25 25136376 PMC4135345

[B7] Bontekoe CJ, Bakker CE, Nieuwenhuizen IM, van der Linde H, Lans H, de Lange D, Hirst MC, Oostra BA (2001) Instability of a (CGG)98 repeat in the Fmr1 promoter. Hum Mol Genet 10:1693–1699. 10.1093/hmg/10.16.1693 11487573

[B8] Brouwer JR, Mientjes EJ, Bakker CE, Nieuwenhuizen IM, Severijnen LA, Van der Linde HC, Nelson DL, Oostra BA, Willemsen R (2007) Elevated Fmr1 mRNA levels and reduced protein expression in a mouse model with an unmethylated Fragile X full mutation. Exp Cell Res 313:244–253. 10.1016/j.yexcr.2006.10.002 17150213 PMC1852528

[B9] Brouwer JR, Huizer K, Severijnen L-A, Hukema RK, Berman RF, Oostra BA, Willemsen R (2008) CGG-repeat length and neuropathological and molecular correlates in a mouse model for fragile X-associated tremor/ataxia syndrome. J Neurochem 107:1671–1682. 10.1111/j.1471-4159.2008.05747.x 19014369 PMC2605773

[B10] Concordet J-P, Haeussler M (2018) CRISPOR: intuitive guide selection for CRISPR/Cas9 genome editing experiments and screens. Nucleic Acids Res 46:W242–W245. 10.1093/nar/gky354 29762716 PMC6030908

[B11] Crawford DC, Acuña JM, Sherman SL (2001) FMR1 and the fragile X syndrome: human genome epidemiology review. Genet Med 3:359–371. 10.1097/00125817-200109000-00006 11545690 PMC4493892

[B12] Darnell JC, Klann E (2013) The translation of translational control by FMRP: therapeutic targets for FXS. Nat Neurosci 16:1530–1536. 10.1038/nn.3379 23584741 PMC3999698

[B13] De Rubeis S, Fernández E, Buzzi A, Di Marino D, Bagni C (2012) Molecular and cellular aspects of mental retardation in the fragile X syndrome: from gene mutation/s to spine dysmorphogenesis. Adv Exp Med Biol 970:517–551. 10.1007/978-3-7091-0932-8_23 22351071

[B14] Denman RB, Sung YJ (2002) Species-specific and isoform-specific RNA binding of human and mouse fragile X mental retardation proteins. Biochem Biophys Res Commun 292:1063–1069. 10.1006/bbrc.2002.6768 11944923

[B15] Diep AA, Hunsaker MR, Kwock R, Kim K, Willemsen R, Berman RF (2012) Female CGG knock-in mice modeling the fragile X premutation are impaired on a skilled forelimb reaching task. Neurobiol Learn Mem 97:229–234. 10.1016/j.nlm.2011.12.006 22202169 PMC3278548

[B16] Dutch-Belgian Fragile X Consortium (1994) Fmr1 knockout mice: a model to study fragile X mental retardation. Cell 78:23–33. 8033209

[B17] Duy PQ, Budimirovic DB (2017) Fragile X syndrome: lessons learned from the most translated neurodevelopmental disorder in clinical trials. Transl Neurosci 8:7–8. 10.1515/tnsci-2017-0002 28400977 PMC5382936

[B18] Entezam A, Biacsi R, Orrison B, Saha T, Hoffman GE, Grabczyk E, Nussbaum RL, Usdin K (2007) Regional FMRP deficits and large repeat expansions into the full mutation range in a new fragile X premutation mouse model. Gene 395:125–134. 10.1016/j.gene.2007.02.026 17442505 PMC1950257

[B19] Evans-Galea MV, Hannan AJ, Carrodus N, Delatycki MB, Saffery R (2013) Epigenetic modifications in trinucleotide repeat diseases. Trends Mol Med 19:655–663. 10.1016/j.molmed.2013.07.007 23953480

[B20] Foote M, Arque G, Berman RF, Santos M (2016) Fragile X-associated tremor/ataxia syndrome (FXTAS) motor dysfunction modeled in mice. Cerebellum 15:611–622. 10.1007/s12311-016-0797-6 27255703 PMC5014696

[B21] Greco CM, Hagerman RJ, Tassone F, Chudley AE, Del Bigio MR, Jacquemont S, Leehey M, Hagerman PJ (2002) Neuronal intranuclear inclusions in a new cerebellar tremor/ataxia syndrome among fragile X carriers. Brain 125:1760–1771. 10.1093/brain/awf184 12135967

[B22] Greco CM, Berman RF, Martin RM, Tassone F, Schwartz PH, Chang A, Trapp BD, Iwahashi C, Brunberg J, Grigsby J, Hessl D, Becker EJ, Papazian J, Leehey MA, Hagerman RJ, Hagerman PJ (2006) Neuropathology of fragile X-associated tremor/ataxia syndrome (FXTAS). Brain 129:243–255. 10.1093/brain/awh683 16332642

[B24] Hagerman R, Hoem G, Hagerman P (2010) Fragile X and autism: intertwined at the molecular level leading to targeted treatments. Mol Autism 1:12. 10.1186/2040-2392-1-12 20858229 PMC2954865

[B25] Hagerman RJ, Berry-Kravis E, Hazlett HC, Bailey DB, Moine H, Kooy RF, Tassone F, Gantois I, Sonenberg N, Mandel JL, Hagerman PJ (2017) Fragile X syndrome. Nat Rev Dis Primers 3:17065. 10.1038/nrdp.2017.65 28960184

[B26] Haify SN, Mankoe RSD, Boumeester V, van der Toorn EC, Verhagen RFM, Willemsen R, Hukema RK, Bosman LWJ (2020) Lack of a clear behavioral phenotype in an inducible FXTAS mouse model despite the presence of neuronal FMRpolyG-positive aggregates. Front Mol Biosci 7:599101. 33381520 10.3389/fmolb.2020.599101 PMC7768028

[B27] Hall DA, Birch RC, Anheim M, Jønch AE, Pintado E, O’Keefe J, Trollor JN, Stebbins GT, Hagerman RJ, Fahn S, Berry-Kravis E, Leehey MA (2014) Emerging topics in FXTAS. J Neurodev Disord 6:31. 25642984 10.1186/1866-1955-6-31 PMC4141265

[B28] Hayward BE, Zhou Y, Kumari D, Usdin K (2016) A set of assays for the comprehensive analysis of FMR1 alleles in the fragile X-related disorders. J Mol Diagn 18:762–774. 10.1016/j.jmoldx.2016.06.001 27528259 PMC5807930

[B29] He CX, Portera-Cailliau C (2013) The trouble with spines in fragile X syndrome: density, maturity and plasticity. Neuroscience 251:120–128. 10.1016/j.neuroscience.2012.03.049 22522472 PMC3422423

[B30] Ho J, Tumkaya T, Aryal S, Choi H, Claridge-Chang A (2019) Moving beyond P values: data analysis with estimation graphics. Nat Methods 16:565–566. 10.1038/s41592-019-0470-3 31217592

[B31] Huber KM, Gallagher SM, Warren ST, Bear MF (2002) Altered synaptic plasticity in a mouse model of fragile X mental retardation. Proc Natl Acad Sci U|S|A 99:7746–7750. 10.1073/pnas.122205699 12032354 PMC124340

[B32] Hunsaker MR (2013) Neurocognitive endophenotypes in CGG KI and Fmr1 KO mouse models of fragile X-Associated disorders: an analysis of the state of the field. F1000Res 2:287. 10.12688/f1000research.2-287.v1 24627796 PMC3945770

[B33] Jacquemont S, Berry-Kravis E, Hagerman R, von Raison F, Gasparini F, Apostol G, Ufer M, Des Portes V, Gomez-Mancilla B (2014) The challenges of clinical trials in fragile X syndrome. Psychopharmacology (Berl) 231:1237–1250. 10.1007/s00213-013-3289-0 24173622 PMC3932172

[B34] Kaiser T, Feng G (2015) Modeling psychiatric disorders for developing effective treatments. Nat Med 21:979–988. 10.1038/nm.3935 26340119 PMC4886231

[B35] Kenneson A, Zhang F, Hagedorn CH, Warren ST (2001) Reduced FMRP and increased FMR1 transcription is proportionally associated with CGG repeat number in intermediate-length and premutation carriers. Hum Mol Genet 10:1449–1454. 10.1093/hmg/10.14.1449 11448936

[B36] Kwok YK, Wong KM, Lo FM, Kong GWS, Moore JK, Wu S, Lam STS, Schermer M, Leung TY, Choy KW (2016) Validation of a robust PCR-based assay for quantifying fragile X CGG repeats. Clin Chim Acta 456:137–143. 10.1016/j.cca.2016.02.027 26947966

[B37] Liu XS, Wu H, Krzisch M, Wu X, Graef J, Muffat J, Hnisz D, Li CH, Yuan B, Xu C, Li Y, Vershkov D, Cacace A, Young RA, Jaenisch R (2018) Rescue of fragile X syndrome neurons by DNA methylation editing of the FMR1 gene. Cell 172:979–992.e6. 10.1016/j.cell.2018.01.012 29456084 PMC6375087

[B38] Lubs HA, Stevenson RE, Schwartz CE (2012) Fragile X and X-linked intellectual disability: four decades of discovery. Am J Hum Genet 90:579–590. 10.1016/j.ajhg.2012.02.018 22482801 PMC3322227

[B39] Ludwig AL, Espinal GM, Pretto DI, Jamal AL, Arque G, Tassone F, Berman RF, Hagerman PJ (2014) CNS expression of murine fragile X protein (FMRP) as a function of CGG-repeat size. Hum Mol Genet 23:3228–3238. 10.1093/hmg/ddu032 24463622 PMC4030777

[B40] Maddalena A, Richards CS, McGinniss MJ, Brothman A, Desnick RJ, Grier RE, Hirsch B, Jacky P, McDowell GA, Popovich B, Watson M, Wolff DJ (2001) Technical standards and guidelines for fragile X: the first of a series of disease-specific supplements to the standards and guidelines for Clinical Genetics Laboratories of the American College of Medical Genetics. Quality Assurance Subcommittee of the Laboratory Practice Committee. Genet Med 3:200–205. 10.1097/00125817-200105000-00010 11388762 PMC3110344

[B41] Mali P, Yang L, Esvelt KM, Aach J, Guell M, DiCarlo JE, Norville JE, Church GM (2013) RNA-guided human genome engineering via Cas9. Science 339:823–826. 10.1126/science.1232033 23287722 PMC3712628

[B42] Martin JP, Bell J (1943) A pedigree of mental defect showing sex-linkage. J Neurol Psychiatry 6:154–157. 10.1136/jnnp.6.3-4.154 21611430 PMC1090429

[B43] McMurray CT (2010) Mechanisms of trinucleotide repeat instability during human development. Nat Rev Genet 11:786–799. 10.1038/nrg2828 20953213 PMC3175376

[B44] Naumann A, Hochstein N, Weber S, Fanning E, Doerfler W (2009) A distinct DNA-methylation boundary in the 5′-upstream sequence of the FMR1 promoter binds nuclear proteins and is lost in fragile X syndrome. Am J Hum Genet 85:606–616. 10.1016/j.ajhg.2009.09.018 19853235 PMC2775827

[B45] Nolin SL, Brown WT, Glicksman A, Houck GE Jr, Gargano AD, Sullivan A, Biancalana V, Bröndum-Nielsen K, Hjalgrim H, Holinski-Feder E, Kooy F, Longshore J, Macpherson J, Mandel J-L, Matthijs G, Rousseau F, Steinbach P, Väisänen M-L, von Koskull H, Sherman SL (2003) Expansion of the fragile X CGG repeat in females with premutation or intermediate alleles. Am J Hum Genet 72:454–464. 10.1086/367713 12529854 PMC379237

[B46] Nolin SL, Glicksman A, Ersalesi N, Dobkin C, Brown WT, Cao R, Blatt E, Sah S, Latham GJ, Hadd AG (2015) Fragile X full mutation expansions are inhibited by one or more AGG interruptions in premutation carriers. Genet Med 17:358–364. 10.1038/gim.2014.106 25210937

[B47] Oberlé I, Rousseau F, Heitz D, Kretz C, Devys D, Hanauer A, Boué J, Bertheas MF, Mandel JL (1991) Instability of a 550-base pair DNA segment and abnormal methylation in fragile X syndrome. Science 252:1097–1102. 10.1126/science.252.5009.1097 2031184

[B48] Qin M, Entezam A, Usdin K, Huang T, Liu Z-H, Hoffman GE, Smith CB (2011) A mouse model of the fragile X premutation: effects on behavior, dendrite morphology, and regional rates of cerebral protein synthesis. Neurobiol Dis 42:85–98. 10.1016/j.nbd.2011.01.008 21220020 PMC3150744

[B49] Reyniers E, Vits L, De Boulle K, Van Roy B, Van Velzen D, de Graaff E, Verkerk AJ, Jorens HZ, Darby JK, Oostra B (1993) The full mutation in the FMR-1 gene of male fragile X patients is absent in their sperm. Nat Genet 4:143–146. 8348152 10.1038/ng0693-143

[B50] Richter JD, Zhao X (2021) The molecular biology of FMRP: new insights into fragile X syndrome. Nat Rev Neurosci 22:209–222. 10.1038/s41583-021-00432-0 33608673 PMC8094212

[B51] Scharf SH, Jaeschke G, Wettstein JG, Lindemann L (2015) Metabotropic glutamate receptor 5 as drug target for fragile X syndrome. Curr Opin Pharmacol 20:124–134. 10.1016/j.coph.2014.11.004 25488569

[B52] Sethna F, Moon C, Wang H (2014) From FMRP function to potential therapies for fragile X syndrome. Neurochem Res 39:1016–1031. 10.1007/s11064-013-1229-3 24346713 PMC4024105

[B53] Suhl JA, Warren ST (2015) Single-nucleotide mutations in FMR1 reveal novel functions and regulatory mechanisms of the fragile X syndrome protein FMRP. J Exp Neurosci 9:35–41. 10.4137/JEN.S25524 26819560 PMC4720182

[B54] Tassone F, Hagerman RJ, Taylor AK, Gane LW, Godfrey TE, Hagerman PJ (2000a) Elevated levels of FMR1 mRNA in carrier males: a new mechanism of involvement in the fragile-X syndrome. Am J Hum Genet 66:6–15. 10.1086/302720 10631132 PMC1288349

[B55] Tassone F, Hagerman RJ, Loesch DZ, Lachiewicz A, Taylor AK, Hagerman PJ (2000b) Fragile X males with unmethylated, full mutation trinucleotide repeat expansions have elevated levels of FMR1 messenger RNA. Am J Med Genet 94:232–236. 10.1002/1096-8628(20000918)94:3<232::AID-AJMG9>3.0.CO;2-H 10995510

[B56] Tekendo-Ngongang C, Grochowsky A, Solomon BD, Yano ST (2021) Beyond trinucleotide repeat expansion in fragile X syndrome: rare coding and noncoding variants in FMR1 and associated phenotypes. Genes (Basel) 12:1669. 10.3390/genes12111669 34828275 PMC8623550

[B57] Todd PK, Oh SY, Krans A, He F, Sellier C, Frazer M, Renoux AJ, Chen K, Scaglione KM, Basrur V, Elenitoba-Johnson K, Vonsattel JP, Louis ED, Sutton MA, Taylor JP, Mills RE, Charlet-Berguerand N, Paulson HL (2013) CGG repeat-associated translation mediates neurodegeneration in fragile X tremor ataxia syndrome. Neuron 78:440–455. 10.1016/j.neuron.2013.03.026 23602499 PMC3831531

[B58] Van Dam D, Errijgers V, Kooy RF, Willemsen R, Mientjes E, Oostra BA, De Deyn PP (2005) Cognitive decline, neuromotor and behavioural disturbances in a mouse model for fragile-X-associated tremor/ataxia syndrome (FXTAS). Behav Brain Res 162:233–239. 10.1016/j.bbr.2005.03.007 15876460

[B59] Wells RD (2009) Mutation spectra in fragile X syndrome induced by deletions of CGG*CCG repeats. J Biol Chem 284:7407–7411. 10.1074/jbc.R800024200 18957433 PMC2658034

[B60] Wenzel HJ, Hunsaker MR, Greco CM, Willemsen R, Berman RF (2010) Ubiquitin-positive intranuclear inclusions in neuronal and glial cells in a mouse model of the fragile X premutation. Brain Res 1318:155–166. 10.1016/j.brainres.2009.12.077 20051238 PMC3086812

[B61] Wilde JJ, Aida T, Del Rosario RCH, Kaiser T, Qi P, Wienisch M, Zhang Q, Colvin S, Feng G (2021) Efficient embryonic homozygous gene conversion via RAD51-enhanced interhomolog repair. Cell 184:3267–3280.e18. 10.1016/j.cell.2021.04.035 34043941 PMC8240950

[B62] Willemsen R, Levenga J, Oostra BA (2011) CGG repeat in the FMR1 gene: size matters. Clin Genet 80:214–225. 10.1111/j.1399-0004.2011.01723.x 21651511 PMC3151325

[B64] Zhao X-N, Usdin K (2018) Timing of expansion of fragile X premutation alleles during intergenerational transmission in a mouse model of the fragile X-related disorders. Front Genet 9:314. 30147707 10.3389/fgene.2018.00314 PMC6096447

