# Supplementary figures and images for: 341 Repeats Is Not Enough for Methylation in a New Fragile X Mouse Model
Source: eNeuro. 2022 Sep 6;9(5):ENEURO.0142-22.2022. doi: 10.1523/ENEURO.0142-22.2022 (PMC9469916; doi:10.1523/ENEURO.0142-22.2022)

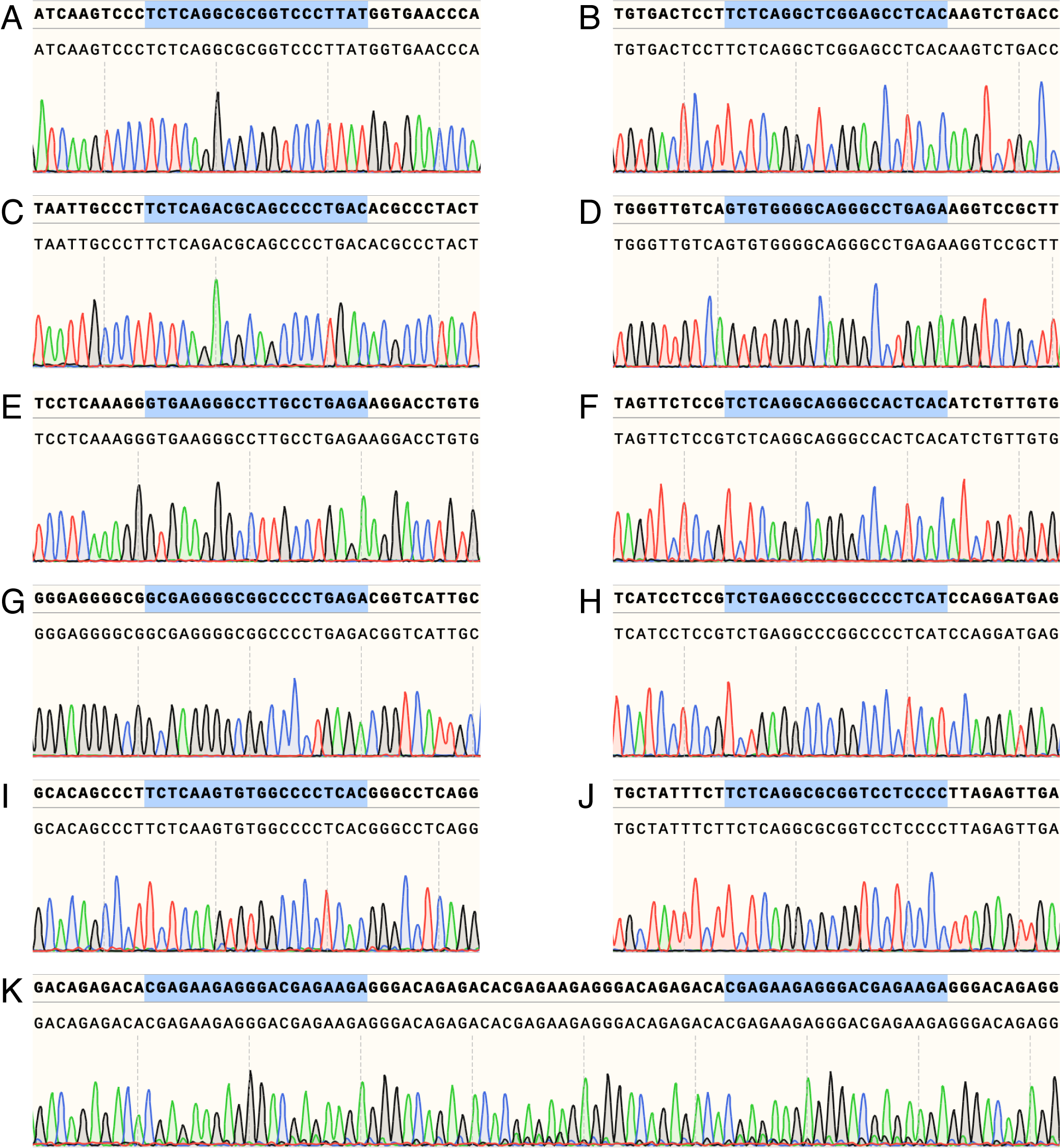

Supplement: Figure 1-1 — Off-target sequencing. A–K. Sequence traces of candidate off-target sites 1-12, respectively (note: off-target sites 11 and 12 are both found in K). Matching off-target sequence is highlighted in blue. The Fmr1hs341 F1 generation displays no sign of any off-target cutting. Download Figure 1-1, TIF file. [file enu-eN-NWR-0142-22-s01.tif]

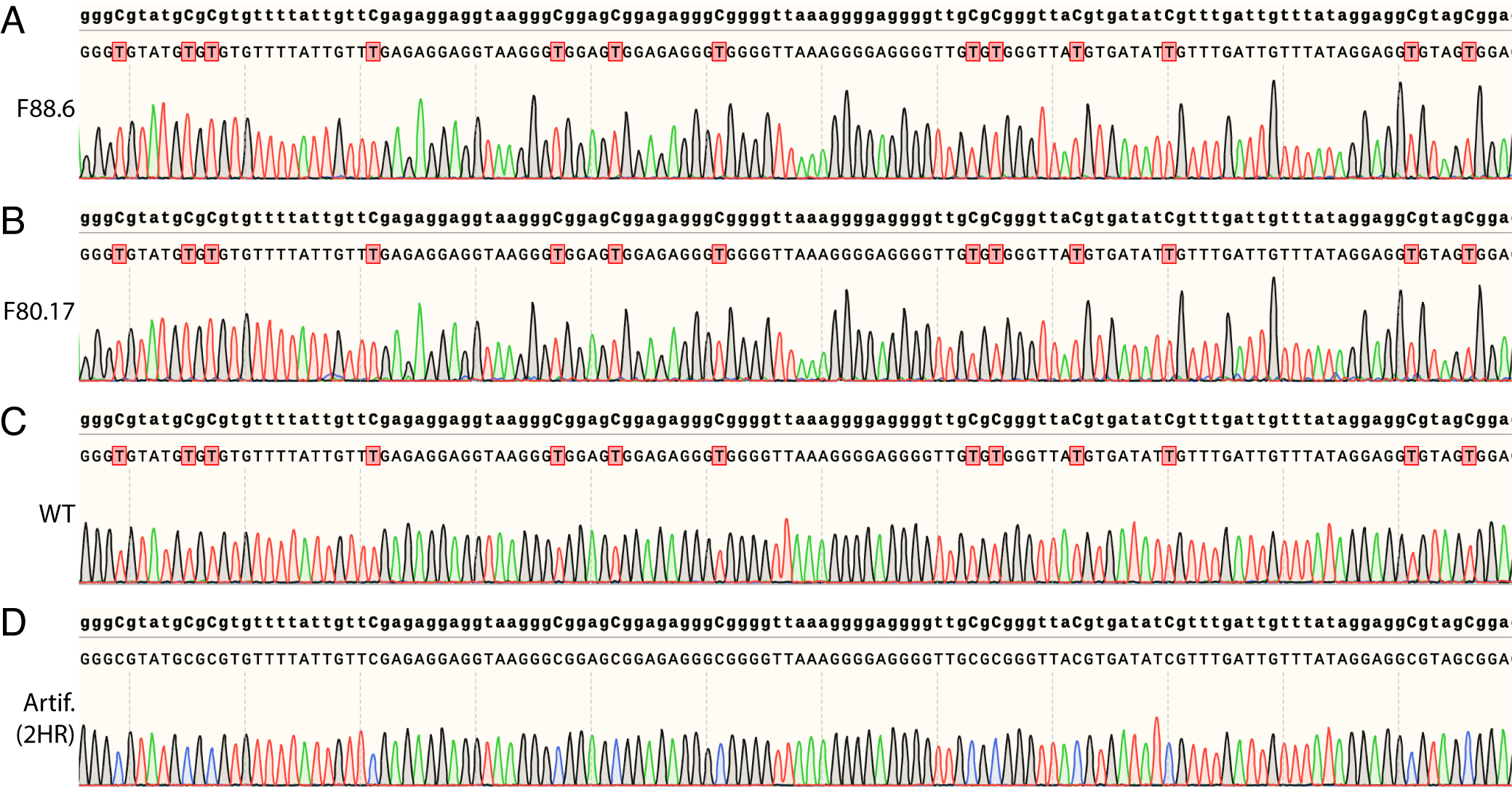

Supplement: Figure 2-1 — Bisulfite sequencing traces. Representative sequence traces of bisulfite analysis. Sequences are aligned to the wild-type sequence where potentially protected cytosines are denoted with an uppercase C. A, Wild-type sibling F88.6 shows no sign of methylation. B, Fmr1hs341 F80.17 shows no sign of methylation. C, Wild-type mouse control shows no sign of methylation. D, Artificially methylated mouse DNA (2 h incubation) demonstrates successful methylation. Download Figure 2-1, TIF file. [file enu-eN-NWR-0142-22-s02.tif]

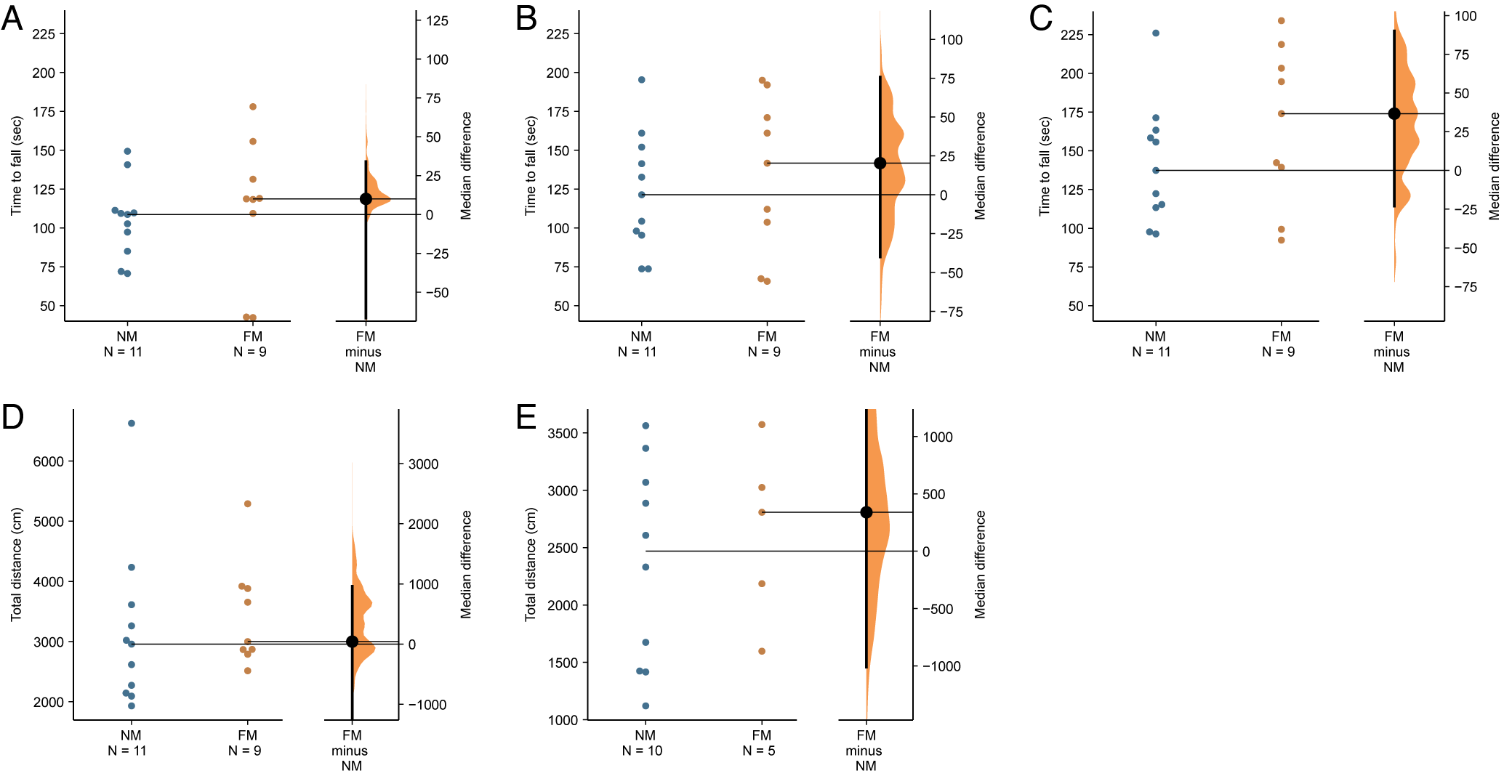

Supplement: Table 2-1 — FMRhs341 does not appear to cause motor deficits. Estimation statistics for behavioral data. All mice were males between 12 and 15 months of age, and age-matched controls were used whenever wildtype siblings were not available. Rightmost data point (FM, full mutation minus NM, no mutation or ∆) represents difference in the medians for effect size. Weighted vertical line indicates 95% confidence interval. Filled curve reflects sampling-error distribution. Generated through Ho et al., 2019 (available at https://www.estimationstats.com/ at time of publication). NM (blue): wildtype; FM (orange): Fmr1hs341. A–C. Time to first fall on the rotarod for mice across three consecutive days compared to wildtype littermates (p-values for: day 1 = 0.252; day 2 = 0.594; day 3 = 0.239) [NM: N = 11; FM: N = 9]. D. Total distance traveled in the open field arena compared to wildtype littermates (p-value = 0.342) [NM: N = 11; FM: N = 9]. E. Total distance traveled in the elevated zero maze apparatus compared to wildtype littermates (p-value = 0.540) [NM: N = 10; FM: N = 5]. Download Table 2-1, TIF file. [file enu-eN-NWR-0142-22-s03.tif]
